# Potential of Pine Biochar to Mitigate Bacterial Hazards Present in Recycled Manure Solids from Dairy Cows

**DOI:** 10.3390/vetsci12010043

**Published:** 2025-01-10

**Authors:** Ana José Pires, Ana Filipa Esteves, Gonçalo Pereira, Catarina Geraldes, Joana Fernandes Guerreiro, Raquel Abreu, Rita Magalhães, Lélia Chambel, Elisabete Silva, David Fangueiro, Manuela Oliveira, Ricardo Bexiga

**Affiliations:** 1 Centre for Interdisciplinary Research in Animal Health (CIISA), Faculty of Veterinary Medicine, University of Lisbon, Av. da Universidade Técnica de Lisboa, 1300-477 Lisbon, Portugal; apires@fmv.ulisboa.pt (A.J.P.); filipaes19@gmail.com (A.F.E.); goncalopereira@fmv.ulisboa.pt (G.P.); cgeraldes@fmv.ulisboa.pt (C.G.); jguerreiro@fmv.ulisboa.pt (J.F.G.); rmsilva@fmv.ulisboa.pt (R.A.); elisabetesilva@fmv.ulisboa.pt (E.S.); ricardobexiga@fmv.ulisboa.pt (R.B.); 2 Associate Laboratory for Animal and Veterinary Sciences (AL4AnimalS), 1300-477 Lisbon, Portugal; 3 Biosystems and Integrative Sciences Institute (BioISI), Faculty of Sciences, University of Lisbon, 1741-016 Lisbon, Portugal; lmchambel@ciencias.ulisboa.pt; 4 LEAF Research Center, Terra Associate Laboratory, Instituto Superior de Agronomia, University of Lisbon, Tapada da Ajuda, 1349-017 Lisbon, Portugal; dfangueiro@isa.ulisboa.pt; 5 cE3c—Centre for Ecology, Evolution and Environmental Changes & CHANGE—Global Change and Sustainability Institute, Faculdade de Ciências, Universidade de Lisboa, Campo Grande, 1749-016 Lisbon, Portugal

**Keywords:** dairy farms, recycled manure solids (RMS), biochar, antimicrobial resistance (AMR), virulence factors, *Escherichia coli*, *Enterococcus*

## Abstract

The use of recycled manure solids (RMS) as cow bedding in dairy farms poses concerns due to its potential to harbor pathogenic and antimicrobial-resistant bacteria. This study evaluated the impact of RMS supplementation with biochar at three concentrations (2.5%, 5%, and 10%) on bacterial counts and on the antimicrobial resistance and virulence profiles of *Escherichia coli* and *Enterococcus* isolates. The results show that biochar supplementation did not promote a significant reduction in bacterial numbers. Overall, there were no significant changes in the isolates’ virulence or resistance profiles, and none of the isolates obtained were classified as high or moderate threats based on their MAR and VIR indexes. The most pathogenic *Enterococcus* isolates found were present in the control samples. A phylogenetic analysis of *E. coli* isolates allowed us to identify phylogroup D strains, predominantly in RMS supplemented with 2.5% and 10% biochar, which are associated with higher virulence and resistance. These findings indicate that the biochar concentrations tested were not effective in significantly reducing the bacterial risks associated with RMS. Further research is needed to evaluate different biochar formulations and concentrations, aiming to optimize its potential use for RMS supplementation.

## Simple Summary

The use of recycled manure solids as bedding for cattle can raise concerns about the spread of bacteria resistant to antibiotics, which could pose risks to both animal and human health. This study investigated whether adding biochar, a material produced by the pyrolysis of organic matter, to manure bedding can reduce harmful bacteria and minimize the risk of antibiotic resistance. The effect of supplementing manure solids with various biochar concentrations was tested in two time periods. The results indicate that the tested concentrations of biochar did not contribute to a reduction in RMS’ bacterial loads nor in the antimicrobial resistance or virulence potential of the bacterial species analyzed. Further studies are necessary to evaluate biochar’s ability to eliminate bacterial agents and determinants from manure solids before proceeding to its broader application in farming systems.

## 1. Introduction

In an era characterized by escalating global demands for animal-based food, the sustainability and safety of dairy farming practices have come under rigorous scrutiny. Of particular concern is the potential contribution of dairy farming to the dissemination of antimicrobial resistance (AMR), which poses significant environmental and health threats worldwide. The misuse of antibiotics in dairy farming creates selective pressure, driving the emergence of antimicrobial-resistant bacteria (ARB). These resistant bacteria can spread through milk, manure, wastewater, and soil to the dairy farm environment, potentially affecting both animals and humans [1,2]. Furthermore, resistance genes can transfer across species and environments via horizontal gene transfer, complicating efforts to address the global challenge of AMR [2]. Therefore, effective management practices are needed, particularly for cattle manure, which may serve as a reservoir for ARB and antimicrobial-resistant genes (ARG) due to intensive milk production practices that generate large volumes of manure rich in bacteria [3]. These bacteria, whose counts usually range between 10^9^ and 10^10^ colony-forming units per gram (CFU/g), may contribute to the environmental reservoir of resistance, underscoring the urgent need for effective mitigation strategies [4,5].

Recycled manure solids (RMS) have gained popularity; they are used as bedding material for dairy cows due to their reduced costs and increased availability. However, the use of RMS raises concerns about bacterial transmission to the animals, including antimicrobial-resistant strains [6]. Unprocessed manure may harbor a diverse array of microbial populations, including potential pathogens, posing risks to animal health. While the practice of using manure as a soil amendment is prevalent worldwide, due to its nutrient-rich composition, high organic matter content, and cost-effectiveness for liquid manure disposal, its use in dairy farming warrants thorough investigation, which is imperative to assess the potential ramifications for the dissemination of AMR [7,8].

To be used as cow bedding in Europe, RMS must adhere to Regulation 1069/2009 set forth by the European Parliament and the Council of the European Union. This regulation establishes a maximum threshold of 1000 *Enterobacteriaceae* CFU/g in manure by-products [4]. Several manure pre-treatment options, including chemical and physical treatments, can be applied to control bacterial levels in this product. However, these methods often fall short in completely eliminating antibiotic residues and pathogens. For instance, chemical treatments frequently fail to remove all antibiotic residues and may introduce additional chemicals into the environment [6,9,10]. On the other hand, solid–liquid separation is effective but expensive and energy-intensive [11]. Composting, despite reducing pathogen load, requires significant time and space and may not eliminate all antibiotic residues. Lastly, anaerobic digestion can leave behind resistant bacteria and genes due to suboptimal destruction conditions [12].

Bedding material can have serious implications in animals’ health since dairy cows typically spend 40 to 65% of their time lying down, promoting direct contact between the mammary gland and the bacteria present in the bedding material, including *Streptococcus* spp. (e.g. *Streptococcus uberis*), coliform species (e.g. *Escherichia coli* and *Klebsiella* spp.)*, Pseudomonas* spp., and *Enterococcus* spp. [13,14], which can be responsible for intra-mammary infections and, therefore, bovine mastitis.

Among the bacterial groups mentioned, the fecal indicator bacteria *E. coli* and enterococci are fundamental targets in studies exploring the use of recycled manure solids (RMS) as cow bedding. Renowned for their robustness and prevalence in fecal matter, these organisms serve as indispensable proxies for assessing microbial contamination levels and associated health hazards inherent to the use of RMS as bedding material in bovine husbandry [15].

Besides being a relevant mastitis pathogen, *E. coli* is also associated with reproductive diseases and calf diarrhea in cattle and may pose a zoonotic risk. This potential is underscored by strains like Enterohemorrhagic *E. coli* (EHEC), which are of major public health concern. EHEC is linked to severe gastrointestinal diseases in humans, including hemorrhagic colitis and the potentially fatal hemolytic uremic syndrome [16]. Cattle serve as the primary reservoir for EHEC, with human infections typically resulting from the consumption of contaminated meat and dairy products or from direct contact with infected animals. Moreover, while EHEC can cause life-threatening infections in humans, cattle, particularly adult cattle, often remain asymptomatic carriers, intermittently shedding the bacteria over extended periods [16,17].

Enterococci are another reliable indicator of fecal contamination, typically being commensal bacteria from the gastrointestinal tracts of animals and humans. They are also able to cause mastitis in cattle as well as nosocomial illnesses in humans, such as bacteremia, endocarditis, and urinary tract infections. Given their wide distribution and resilience in the environment, enterococci serve as reliable indicators of fecal contamination, playing a pivotal role in AMR surveillance systems for both human and animal health [18,19,20,21].

To reduce udder exposure to pathogenic bacteria and the incidence of associated infections, cost-effective methods are needed to control RMS microbiota [4,6,22]. Several studies have evaluated the efficacy of different compounds, including biochar, in reducing ARB in animals’ manure. Biochar, a by-product of the pyrolysis of agricultural waste or other types of biomasses, has been described as a cost-effective product for use as an amendment of animal manure, including in dairy farms [23,24,25,26,27]. Biochar represents a promising alternative due to its distinctive properties [28]. Its high surface area and porous structure enable it to adsorb a wide range of substances, including heavy metals, antibiotics, and organic pollutants, and it also has a noteworthy impact on microbial communities within manure [25,27,29]. Specifically, biochar pores can serve as habitats for beneficial microorganisms, promoting their growth and activity while leading to the suppression of pathogenic organisms, thereby improving soil health and reducing disease transmission risks [25,27].

Before addressing its widespread use in dairy farms, RMS supplemented with biochar must be properly characterized concerning its microbial traits with the aim of evaluating its efficacy in inhibiting the dissemination of pathogenic and antimicrobial-resistant bacteria. As such, an incubation experiment with RMS supplemented with three different concentrations of biochar was performed over one month in two distinct time periods. To assess the impact of biochar supplementation on bacterial populations, RMS-supplemented samples from each condition (2.5%, 5%, and 10% biochar) were tested for the presence of *E. coli* and enterococci. Then, the antimicrobial susceptibility and virulence profiles of the isolated strains were analyzed to evaluate any changes attributable to the biochar treatments.

## 2. Materials and Methods

### 2.1. Incubation Experiment

An incubation experiment was conducted using fresh RMS from a commercial dairy farm in Portugal, obtained by mechanical separation. RMS was collected from the same commercial dairy farm at two time periods, namely April and June 2022 (Day 0). In the 30 days of the trials performed in each time period, the relative environmental humidity in the humid period (April–May) was much higher (71.5%) than in the dry period (June–July, 56.3%). The highest overall variations in both temperature and humidity were observed in the dry period (12.6 °C and 56.0% as opposed to 9.5 °C and 43.0% variations in the humid period) (https://www.ipma.pt/pt/index.html (accessed on 2 November 2024)). To avoid variability due to animal differences, only manure from one farm was used in both assays. The manure was collected directly after mechanical separation, mixed thoroughly to ensure homogeneity, and then divided into the appropriate experimental groups.

Subsequently, to test the influence of biochar supplementation on this product’s microbiota, the obtained RMS was divided into five groups as follows: (1) non-supplemented RMS (negative control); (2) RMS supplemented with 10% H_2_SO_4_ (positive control, selected due to its known effectiveness in reducing bacterial counts through acidification); (3) RMS supplemented with 2.5% biochar (2.5B); (4) RMS with 5% biochar (5B); and (5) RMS with 10% biochar (10B) (percentages by weight (*w*/*w*)). Each group consisted of a total of 15 kg, representing three 5 kg replicates of RMS. Replicates from all groups were placed in identical and naturally ventilated containers and stored at ambient temperature for 30 days for each trial period: April–May 2022, corresponding to a more humid and cooler time period, and June–July 2022, a warmer and drier time period. The containers’ contents were mixed every other day to ensure aeration.

The biochar used in this study was produced in Portugal, being obtained by pyrolysis of pine; however, the physicochemical characteristics (e.g., porosity, acidity, and particle size) were not disclosed by the biochar-producing company at the time of the study. The biochar used in all assays was from the same production batch.

### 2.2. Sample Collection

Ten grams of samples of RMS from all experimental groups was collected on days 0, 5, 15, and 30 of the two incubation trials. Sampling time points were selected to represent both the immediate and longer-term responses of the microbial populations to RMS supplementation. Day 0 represents the baseline, allowing for an initial assessment of the microbiota before any treatments had time to produce any effect. Day 5 was chosen to capture early microbial responses to biochar supplementations, as bacterial populations often begin to adjust within the first few days. Day 15 represents a midpoint, providing insights into the sustained effects of the treatments and any ongoing shifts in the microbial community. Finally, Day 30 was selected as the endpoint to assess the long-term impacts of the biochar supplementations, allowing for the evaluation of the stability of microbial changes over a one-month incubation period. These timepoints were intended to provide a comprehensive overview of bacterial growth and adaptation throughout the study.

Ten grams of biochar-supplemented samples, and those from the negative control, were suspended in 10 mL of saline solution and homogenized using a stomacher to obtain a 1:1 suspension that could be used for further processing. Subsequently, ten-fold serial dilutions (10^−1^, 10^−2^, 10^−3^, 10^−4^, 10^−5^, and 10^−6^) in saline solution were carried out. After that, 100 μL of the suspensions were inoculated on the surface of MacConkey Agar (MAC) (VWR, Leuven, Belgium) and Slanetz and Bartley Agar (SB) (AppliChem, Darmstadt, Germany) plates using sterile glass beads. After 48 h of incubation at 37 °C, quantification of *Enterobacteriaceae* (total colonies on MAC) and of enterococci (total small round burgundy colonies on SB) was performed and expressed as CFU/g of bedding sample.

Then, up to four colonies presumptively identified as *E. coli* (lactose-fermenting colonies surrounded by a halo of bile salt precipitation on MAC) and as enterococci were collected from the plates corresponding to each experimental condition and replicate and inoculated onto Brain Heart Infusion (BHI) (VWR) agar plates, followed by incubation at 37 °C for 24 h.

All isolates were stored in buffered peptone water supplemented with glycerol (20%) (VWR) at −20 °C throughout the duration of the study.

### 2.3. Isolates’ Identification and Molecular Characterization

For all molecular assays, a negative control (sterile PCR water) was included, and 10% of independent replicas were performed to validate and assess the results’ reproducibility.

#### 2.3.1. Enterococci

The phenotypical identification of the presumptive enterococci isolates was performed as previously described [30]. Each isolate was inoculated on Bile Esculin (BE) agar (Scharlau, Barcelona, Spain) plates, a selective and differential medium for the isolation and identification of *Enterococcus* spp., and incubated at 37 °C for 42 h. Gram staining and catalase testing were performed for all the isolates that produced dark brown to black colonies in BE agar, aiming to detect Gram-positive and catalase-negative isolates, which were presumptively identified as *Enterococcus* spp. [30].

For molecular identification, the DNA from these isolates was obtained using the boiling method [31]. DNA purity and concentration were assessed using a Nanodrop spectrophotometer (ThermoFisher Scientific, Waltham, MA, USA), followed by sample dilution to achieve a final DNA concentration of 50 ng/μL.

Genus identification followed an adaptation of the method described by Ke et al. (1999) [32]. The reaction mixtures, with a total volume of 25 μL, consisted of 12.5 μL of Supreme NZYTaq II 2x Green Master Mix (NZYTech, Lisbon, Portugal), 1 μM of each of the primers Ent1 and Ent2 (StabVida, FCT/UNL, Caparica, Portugal) (Table 1), and 1 μL of DNA [50 ng/μL] [32]. Amplification was conducted using a XTender^96^ (VWR) thermocycler under the following conditions: initial denaturation at 94 °C for 3 min, followed by 35 cycles of denaturation at 94 °C for 60 s, annealing at 48 °C for 60 s, extension at 72 °C for 60 s, and a final extension at 72 °C for 5 min. All PCR products were separated by agarose gel electrophoresis (1.3%, *w*/*v*) in 1x TBE buffer (NZYTech) supplemented with GreenSafe Premium (NZYTech). The electrophoresis process was conducted at 90 V for 1 h, and the outcomes were visualized using the ChemiDoc™ Gel Imaging System (Bio-Rad, San Diego, CA, USA).

Molecular identification of enterococci species was carried out using a multiplex PCR protocol adapted from [33]. The following species were targeted: *Enterococcus faecium* (primers FM1 and FM2), *Enterococcus faecalis* (FL1 and FL2), *Enterococcus hirae* (HI1 and HI2), *Enterococcus durans* (DU1 and DU2), *Enterococcus casseliflavus* (CA1 and CA2), and *Enterococcus cecorum* (CE1 and CE2) (Table 1). The following positive controls were used: *E. faecalis* ATCC 29212^®^, *E. faecium* CCUG 36804^®^, *E. hirae* ATCC 10541^®^, *E. durans* DSMZ 20633^®^, *E. cecorum* DSMZ 20682^®^, and *E. casseliflavus* DSMZ 20680^®^. A negative control (sterile PCR water) was included in each reaction. Additionally, 10% of independent replicates was tested for result validity and reproducibility.

For PCR reactions targeting *E. faecium* and *E. faecalis*, 25 μL reaction mixtures were prepared with 12.5 μL Supreme NZYTaq II 2x Green Master Mix (NZYTech), 1.25 μM of each primer, and 1 μL of DNA (50 ng/μL). Amplification was performed with the following cycle conditions: initial denaturation at 95 °C for 5 min, followed by 35 cycles of denaturation at 95 °C for 60 s, annealing at 54 °C for 1 min, and extension at 72 °C for 1 min, with a final extension at 72 °C for 10 min. For the identification of *E. hirae*, *E. durans*, *E. cecorum*, and *E. casseliflavus*, PCR mixtures containing 12.5 μL of Supreme NZYTaq II 2x Green Master Mix, 0.75 μM of each primer, and 2 μL of DNA (50 ng/μL) were used under similar amplification conditions, but we applied 30 cycles and an annealing temperature of 55 °C and final extension for 7 min [33].

Genomic fingerprinting of enterococci was performed using a (GTG)_5_ primer-based PCR method adapted from [34]. PCR reaction mixture contained 1x reaction buffer, 3 μM MgCl_2_, 0.2 μM of each deoxynucleotide triphosphate, 2 μM of the primer, 0.06 U of Taq (Invitrogen, Waltham, MA, USA), and 100 ng of DNA. Amplification conditions included initial denaturation at 94 °C for 4 min, followed by 40 cycles of denaturation at 94 °C for 60 s, annealing at 40 °C for 2 min, extension at 72 °C for 2 min, a final extension at 72 °C for 10 min [34].

PCR products were separated by agarose gel electrophoresis (1.5%, *w*/*v*) in 0.5x TBE buffer, stained with GreenSafe Premium (NZYTech), and visualized using the ChemiDoc™ Gel Imaging System (Bio-Rad, Image Lab, Version 6.1.0). Genomic profiles were analyzed using hierarchical clustering with BioNumerics^®^ 6.6 (Applied Maths, Kortrijk, Belgium).

#### 2.3.2. *E. coli*

The presumptive identification of *E. coli* isolates was performed by Gram staining (Gram-negative non-sporulating bacillus), an oxidase test (oxidase-negative), and IMViC testing, which followed an adapted protocol derived from Fernandes et al. (2022) focusing on testing for indole, motility, Voges–Proskauer, and citrate utilization [37]. Isolates that exhibited a positive reaction for indole and motility, along with a negative reaction for Voges–Proskauer and citrate, were presumptively identified as *E. coli* and subjected to genomic fingerprinting and phylogenetic grouping.

DNA from the *E. coli* isolates was obtained utilizing the boiling method [31]. DNA purity and concentration were assessed using a Nanodrop spectrophotometer (ThermoFisher Scientific), and all samples were diluted to achieve a final DNA concentration of 50 ng/μL. Isolates’ genomic fingerprinting was accomplished through ERIC-PCR following the methodology outlined by Silva et al. (2009). The PCR mixture consisted of 10 μL of sterile PCR water, 12.5 μL of MasterMix (NZYTech), 0.5 μL of the ERIC2 primer (Table 1) (Stabvida), and 2 μL of template DNA [50 ng/μL] for a final volume of 25 μL. Amplification was conducted using an XTender⁹⁶ (VWR) thermocycler under the following conditions: initial denaturation at 95 °C for 7 min, followed by 30 cycles of denaturation at 90 °C for 30 s, annealing at 52 °C for 60 s, extension at 72 °C for 8 min, and a final extension at 72 °C for 16 min [35]. All products were separated by agarose gel electrophoresis (1.5%, *w*/*v*) in 0.5x TBE buffer (NZYTech) supplemented with GreenSafe Premium (NZYTech). Electrophoresis was conducted at 70 V for 2 h.

As for enterococci, all electrophoresis outcomes were visualized using the ChemiDoc™ Gel Imaging System (Bio-Rad), and fingerprinting results were assessed using BioNumerics^®^ 6.6 (Applied Maths) as previously described.

Phylogenetic grouping of the *E. coli* isolates was performed using primers focusing on the *gadA*, *chuA*, and *yjaA* genes and the DNA fragment TSPE4-*C2* (Stabvida) (Table 1) [36]. PCR was conducted in a reaction volume of 20 μL, comprising 0.4 μL of each primer with a final concentration of 1 μM, 10 μL of Master Mix (NZYTech), 5.8 μL of sterile PCR water, and 1 μL of bacterial DNA [200 ng/μL]. Amplification was conducted using an XTender⁹⁶ (VWR) thermocycler under the following conditions: initial denaturation at 94 °C for 4 min, followed by 30 cycles of denaturation at 94 °C for 30 s, annealing at 65 °C for 30 s, extension at 72 °C for 30 s, and a final extension at 72 °C for 5 min [36]. Three positive controls (*E. coli* strain J96 belonging to phylogenetic group B2, *E. coli* strain KS52 belonging to phylogenetic group A, and *E. coli* strain 22.8 D belonging to phylogenetic group D) were included.

PCR products underwent separation through agarose gel electrophoresis (2%, *w*/*v*) in 0.5x TBE buffer (NZYTech) supplemented with GreenSafe Premium (NZYTech). Electrophoresis was carried out at 70 V for 2 h, and the results were observed as previously described.

### 2.4. Antimicrobial Susceptibility Testing

Antimicrobial susceptibility testing of the *Enterococcus* spp. and *E. coli* representative isolates selected based on PCR fingerprinting was performed using the disk diffusion method following the Clinical and Laboratory Standards Institute (CLSI) standards [38,39]. To ensure the validity and reproducibility of the assays, 10% independent replicas were performed. Reference strains *E. coli* ATCC 25922, *Staphylococcus aureus* ATCC 25923, and *E. faecalis* ATCC 29212 were used as control strains.

The antibiotics tested were selected based on their frequent use on dairy farms and in human medicine and included compounds from several antibiotic classes. For *Enterococcus* spp., the antibiotics tested included penicillins (ampicillin, 10 μg; amoxicillin–clavulanic acid, 20/10 μg); glycopeptides (vancomycin, 30 μg); tetracyclines (oxytetracycline, 30 μg); aminoglycosides (high-dose gentamicin, 120 μg); and fluoroquinolones (enrofloxacin, 5 μg) (Oxoid Limited^®^, Hampshire, UK). For *E. coli*, the antibiotics tested included penicillins (ampicillin, 10 μg; amoxicillin–clavulanic acid 20/10 μg); tetracyclines (oxytetracycline, 30 μg); sulphonamides (trimethoprim/sulfamethoxazole, 1.25/23.75 μg); fluoroquinolones (enrofloxacin, 5 μg); and cephalosporins (ceftiofur, 30 μg) (Oxoid Limited^®^).

Bacterial suspensions, with a turbidity equivalent to 0.5 on the McFarland scale (equivalent to approximately 1.5 × 10^8^ CFU/mL), were inoculated using the lawn technique on the surface of Mueller–Hinton agar (Oxoid Limited^®^) plates. Antibiotic disks were then placed on the agar surface, and plates were incubated for 18 h at 36 °C, except for vancomycin testing plates, which were incubated for 24 h at the same temperature. After incubation, the diameters of the inhibition halo around the disks were measured, and results were interpreted according to CLSI guidelines M31 A3 [40], VET09 [39], and M100 [38].

The Multiple Antibiotic Resistance (MAR) index for each isolate was calculated according to Singh et al. (2017), considering the number of antimicrobials to which the isolates were resistant to divided by the number of antimicrobials tested. An average of each MAR was then calculated for the isolates from each treatment condition [41].

### 2.5. Virulence Assays

The phenotypic virulence profile of all representative isolates was established by assessing their ability to produce hemolysin, gelatinase, biofilm, DNase, proteinase, and lecithinase using specific media following the procedures used by Fernandes et al. (2022) [37]. Positive and negative controls, along with 10% independent replicas, were also tested.

Hemolysin production was evaluated on Columbia agar medium supplemented with 5% sheep blood (bioMérieux, Marcy-l’étoile, France) using *S. aureus* ATCC^®^ 25923 as positive control and *E. coli* ATCC^®^ 25922 as negative control. A positive reaction was indicated by the formation of a halo around the colonies after incubation at 37 °C for 72 h.

Gelatinase activity was assessed using nutrient gelatin agar (Oxoid Limited ^®^), with *Pseudomonas aeruginosa* ATCC^®^ 27853 as positive control and *E. coli* ATCC^®^ 25922 as negative control. After an incubation period of 48 h at 37 °C, the cultures were refrigerated for 30 min at 4 °C, with liquefaction of the medium being considered a positive result.

Biofilm formation was evaluated in BHI agar medium (VWR) supplemented with 0.8% Congo Red (Sigma-Aldrich, St. Louis, MO, USA) and 5% sucrose (Millipore Sigma-Aldrich, ON, Canada), with *E. faecium* ATCC® 29212 being used as positive control and *E. coli* ATCC^®^ 25922 as negative control. Positive reactions were characterized by the formation of colonies ranging from brown to black after incubation at 37 °C for 72 h.

DNase activity was assessed in DNase agar (VWR) supplemented with 0.01% toluidine blue (Merck KGaA, Darmstadt, Germany), using *S. aureus* ATCC^®^ 25923 as positive control and *E. coli* ATCC^®^ 25922 as negative control. A pink halo around the colonies after incubation at 37 °C for 72 h indicated a positive reaction.

Proteinase activity was evaluated in Skim Milk Agar (VWR), testing *P. aeruginosa* ATCC^®^ 27853 as positive control and *S. aureus* ATCC^®^ 29213 as negative control. The presence of a transparent halo around the colonies after 48 h of incubation at 37 °C denoted a positive reaction.

Lecithinase activity was assessed in Tryptic Soy Agar (VWR) supplemented with 10% egg yolk emulsion (VWR), with *P. aeruginosa* ATCC^®^ 27853 being used as positive control and *E. coli* ATCC^®^ 25922 as negative control. A positive reaction resulted in the formation of a white precipitation halo around the colonies after incubation at 37 °C for 72 h.

The virulence index (VIR) for each isolate was determined by dividing the sum of all positive virulence phenotypes exhibited by the isolates by the total number of virulence factors tested [41]. An average of each VIR was then calculated for each treatment.

Finally, isolates were classified according to their threat level. According to the MAR and VIR classification system created by Singh et al. (2017) [41], isolates can be categorized as a high threat if they exhibit a MAR index ≥ 0.30 and a VIR index ≥ 0.50; as a moderate threat if the MAR index is <0.30 and the VIR index is ≥0.50; as a low threat if the MAR index is ≥0.30 and the VIR index is <0.50; and, finally, as no threat if the MAR is <0.30 and the VIR index is <0.50.

### 2.6. Data Analysis

To evaluate the effect of biochar supplementation on bacterial counts, specifically of *Enterococcus* spp. and *Enterobacteriaceae*, a comprehensive statistical analysis was performed using SAS (version 9.4, SAS Institute Inc., Cary, NC, USA) [42]. The bacterial counts were first adjusted by adding a constant of 1, and then they were log-transformed to normalize the data. A linear mixed-effects model (PROC MIX, SAS) was employed to account for the repeated measures design of the study. The model included treatment (C−, C+, 2.5%, 5%, and 10%) and time (0, 5, 15, and 30) as fixed effects, with replicate as a random effect. This model allowed us to account for the hierarchical structure of the data and the correlation between repeated measurements on the same replicate. The Kenward–Roger method was used for degrees of freedom calculation to improve the accuracy of the fixed-effect tests. Least squares means (LS-means) were computed for each treatment and time point, and pairwise comparisons were adjusted using the Tukey method to control the family-wise error rate. The covariance structures used were the ones resulting in the lowest Akaike information criteria, CS (compound symmetry) for *Enterococcus* and AR (1) (first-order autoregressive) for *Enterobacteriaceae*. The significance of biochar treatments in the isolates’ antibiotic susceptibility and virulence profiles was determined using a generalized linear mixed model with a logit link and binary distribution for the outcome variable, following the PROC GLIMMIX procedure in SAS. Manual backward elimination, guided by a *p*-value threshold of 0.157 as suggested by Heinze and Dunkler (2017), was conducted without initially filtering individual variables to establish the final models [43]. The variable “condition” was included in the model regardless of its statistical significance. In the final models, differences were considered significant when *p* ≤ 0.05. The distribution of the different *E. coli* phylogenetics groups was analyzed with Chi-Square Test of Independence (PROC FREQ).

Given that the MAR and virulence indexes could only assume one of the following values (0.10, 0.11, 0.12, 0.18, 0.19, 0.23, and 0.33 for the MAR index and 0.17, 0.19, 0.22, 0.23, 0.26, 0.32, and 0.37 for the virulence index), they were analyzed as ordinal response variables and fitted to cumulative logistic regression models (PROC LOGISTIC).

## 3. Results

### 3.1. Bacteria Quantification

The raw data regarding bacterial counts in both CFU/g and log units are available in Tables S2 and S3 in the Supplementary Files.

**Figure 1:**
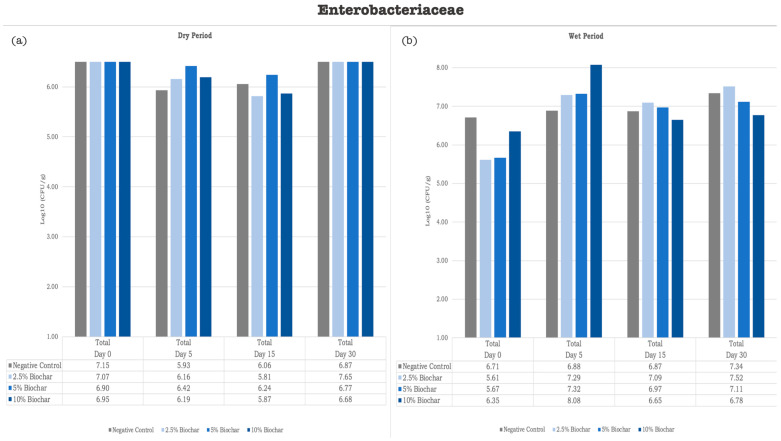
Graphic representation of *Enterobacteriaceae* quantification [Log10(CFU/g)]. (**a**) Bacteria quantification [Log10(CFU/g)] in MAC inoculated with samples collected during assay performed in dry period; (**b**) bacteria quantification [Log10(CFU/g)] in MAC inoculated with samples collected during assay performed in wet period. Logarithmic values were calculated based on average CFU obtained from three replicates for each treatment and time point.

Results from bacterial quantification on the RMS samples supplemented with biochar (2.5%, 5%, and 10%) did not show significant differences from those from the control group. The bar charts in Figure 1 illustrate the mean *Enterobacteriaceae* counts across different treatments and time points. As depicted, *Enterobacteriaceae* counts showed different trends. In the assay performed during the dry period (June–July), a log decrease was observed by Day 30 in the negative control (of 0.28 log units), as well as in the RMS supplemented with 5% biochar (of 0.13 log units) and in the RMS supplemented with 10% biochar (of 0.27 log units); on the contrary, a log increase was observed by Day 30 in the RMS samples supplemented with 2.5% biochar (of 0.58 log increase). The same tendency was observed in the assay performed during the wet period (April–May) by Day 30, in which all RMS samples (including the negative controls and the biochar-supplemented samples) showed an increase in *Enterobacteriaceae* log units, ranging from 0.43 (for the samples supplemented with 10% biochar) to 1.91 (for the samples supplemented with 2.5% biochar).

**Figure 2:**
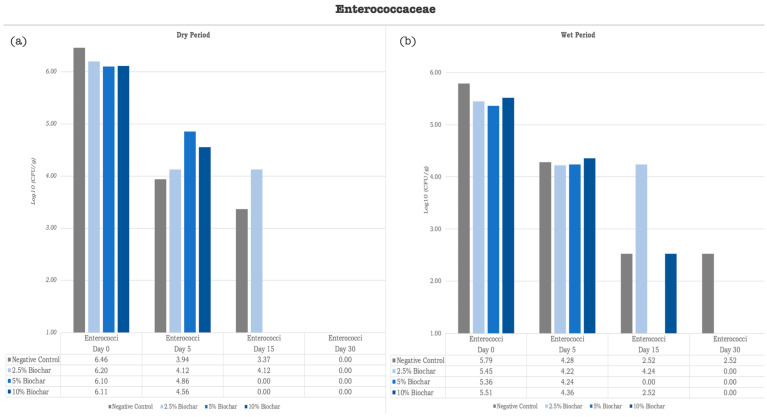
Graphic representation of *Enterococcaceae* quantification [Log10(CFU/g)]. (**a**) Bacteria quantification [Log10(CFU/g)] in SB inoculated with samples collected during assay performed in dry period; (**b**) bacteria quantification [Log10(CFU/g)] in SB inoculated with samples collected during assay performed in wet period. Logarithmic values were calculated based on average CFU obtained from three replicates for each treatment and time point.

Figure 2 illustrates the mean *Enterococcaceae* counts across different treatments and time points. By Day 30, a decrease in *Enterococcaceae* log units was observed in the two assays for all the conditions tested. In the assay performed during the dry period (June–July), the log decreases observed ranged between 6.10 log in the RMS samples supplemented with 5% biochar and 6.46 log in the negative control samples, while in the assay performed during the wet period (April–May), the log decreases observed ranged between 3.27 log in the negative control samples and 5.51 log in the RMS samples supplemented with 10% biochar.

As seen in Table 2, significant reductions in *Enterococcus* spp. counts were observed over time. However, the effects of biochar supplementation at 2.5%, 5%, and 10% on reducing *Enterococcus* spp. counts were not statistically significant. Similarly, for *Enterobacteriaceae*, no significant differences were found between biochar treatments and the negative control for either bacterial group.

### 3.2. Enterococcus Identification and Molecular Characterization

From the incubation assay, a total of 103 presumptive enterococci isolates were obtained from all RMS treatment groups during the assay performed in the wet period, from which only 31 were confirmed as belonging to the genus *Enterococcus* by phenotypic tests. Similarly, during the assay performed in the dry period, 109 presumptive enterococci isolates were collected from all RMS treatment groups, with 22 being phenotypically confirmed as belonging to the genus *Enterococcus*. Of these 53 isolates, 50 were confirmed as belonging to the genus *Enterococcus* by PCR and further identified to the species level.

In this study, the identification of *Enterococcus* isolates at species level, including *E. faecium, E. faecalis*, *E. hirae*, and *E. durans*, was achieved using specific primers designed for each target species. However, for *E. cecorum* and *E. casseliflavus,* the size of the PCR products obtained did not match the expected sizes for either species (371 bp and 288 bp, respectively). Subsequently, the PCR products were subjected to a sequencing analysis, and the isolates’ identification as *E. gallinarum* was confirmed using the BLAST (Basic Local Alignment Search Tool) algorithm, as seen in Table S1 in the Supplementary Files.

As observed in Table 3, *E. faecium* was detected only in the wet period, with 16% being found in the negative control samples, 3% in the samples of RMS supplemented with 5% biochar, and 6% in the samples of RMS supplemented with 10% biochar. *E. faecalis* appeared in 6% of the samples of RMS supplemented with 5% biochar during the wet period. *E. hirae* showed a 32% prevalence in the samples of RMS supplemented with 2.5% biochar during the dry period and also appeared in 11% of the samples with 5% biochar. *E. gallinarum* was identified in 13% of the samples of RMS supplemented with 2.5% biochar and 16% in the samples of RMS supplemented with 10% biochar during the wet period, whereas it showed a 16% prevalence in the negative control during the dry period. *Enterococcus* sp. had a higher presence in the wet period, with 10% being found in the negative control samples and 6% in the samples of RMS supplemented with 2.5% and 5% biochar. In the dry period, it appeared in 11% of the negative control and 11% of the samples with 10% biochar. The samples with fewer occurrences of the *Enterococcus* species known for their elevated health risks, *E. faecium* and *E. faecalis*, were the samples of RMS supplemented with 2.5% biochar during the dry period.

The (GTG)_5_ fingerprinting analysis allowed to compare the profiles of *Enterococcus* isolates and select 40 representative isolates for further tasks. Using a reproducibility cut-off of 88.5%, the isolates with identical fingerprinting profiles, obtained from the samples collected in the same period, treatment group, and sampling day, were excluded.

**Figure 3:**
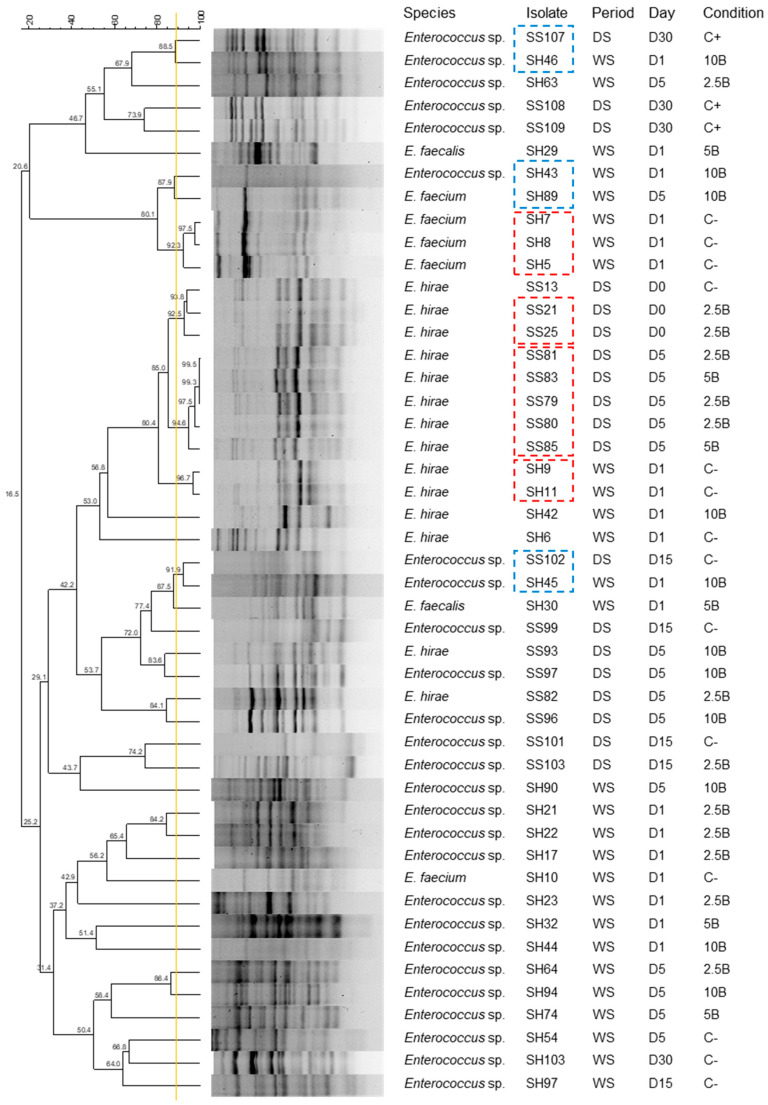
Dendrogram of *Enterococcus* isolates obtained using (GTG)_5_ molecular fingerprinting. Isolates within red dashed rectangles (SH5, SH7, SH9, SS21, SS79, SS80, and SS83) were excluded due to their clonal similarity and matching collection parameters. However, isolates within blue dashed rectangles, despite being clones, were not excluded as they were collected in different periods, days, or conditions. Yellow line—cut-off line of 88.5% (reproducibility value). D—day of sample collection; C+—positive control; C−—negative control; 2.5B—RMS supplemented with 2.5% biochar; 5B—RMS supplemented with 5% biochar; 10B—RMS supplemented with 10% biochar; WS—wet period DS—dry period.

The dendrogram (Figure 3) revealed clonal relationships among the *Enterococcus* isolates from the RMS samples supplemented with biochar under different conditions. Isolates from the wet period (WS) and dry period (DS) were analyzed for clonal similarity. Globally, there are isolates with different profiles indicating a level of genetic diversity, with seven clusters representing clonal relationships (≥ 85% similarity). 

### 3.3. E. coli Identification and Molecular Characterization

The sample culture yielded 192 and 170 presumptive *E. coli* isolates from assays performed during the wet and dry periods, respectively. Using the phenotypic identification method described in the Materials and Methods Section, 28 and 44 isolates were confirmed as *E. coli* from the wet and dry periods, respectively. This discrepancy between presumptive and confirmed isolates highlights the challenges of accurate identification. The method involved an initial selection based on colony morphology on MacConkey agar, followed by biochemical tests. However, false positives can occur due to non-target bacteria exhibiting similar phenotypic traits [44].

Using ERIC fingerprinting, it was possible to compare the profiles of the 72 *E. coli* isolates and select 42 representatives for further analysis based on a reproducibility cut-off of 68.8%.

**Figure 4:**
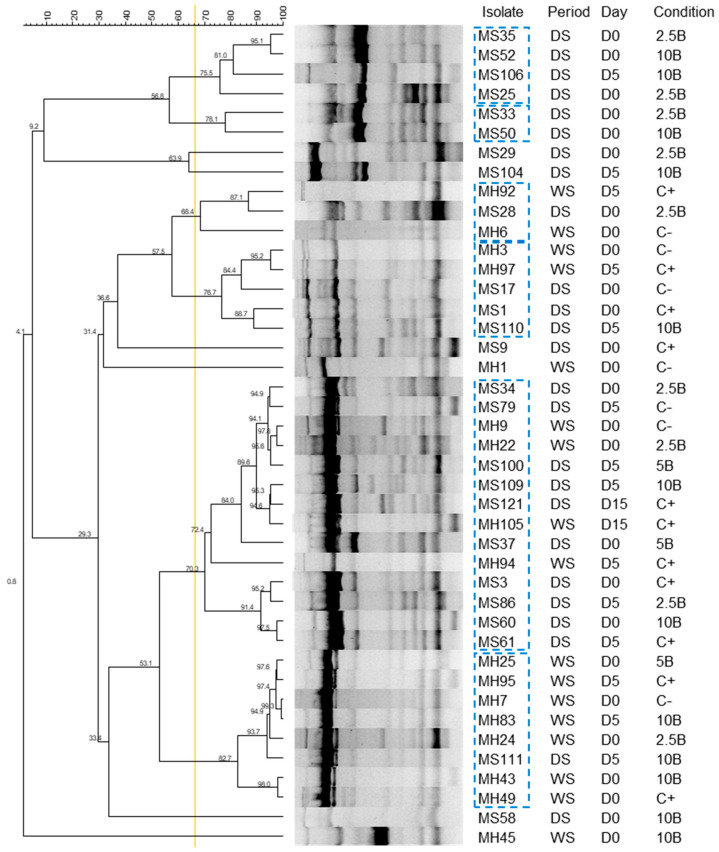
Dendrogram of representative *E. coli* isolates obtained using REP–PCR molecular fingerprinting. Isolates within blue dashed rectangles, despite being clones, were not excluded as they were collected in different periods, days, or conditions. Yellow line—cut-off line of 68.8%. D—day of sample collection; C+—positive control; C−—negative control; 2.5B—RSM supplemented with 2.5% biochar; 5B—RSM supplemented with 5% biochar; 10B—RSM supplemented with 10% biochar; WS—wet period; DS—dry period.

The dendrogram (Figure 4) illustrates the relationships among *E. coli* isolates from RMS samples. Some genetic diversity is observed, but there are also major clusters with ≥68.8% similarity, containing isolates from different periods and biochar supplementation.

The results from the fingerprinting assay allowed us to exclude isolates with the same fingerprinting profile that were obtained from samples collected in the same period, treatment group, and sampling day.

**Figure 5:**
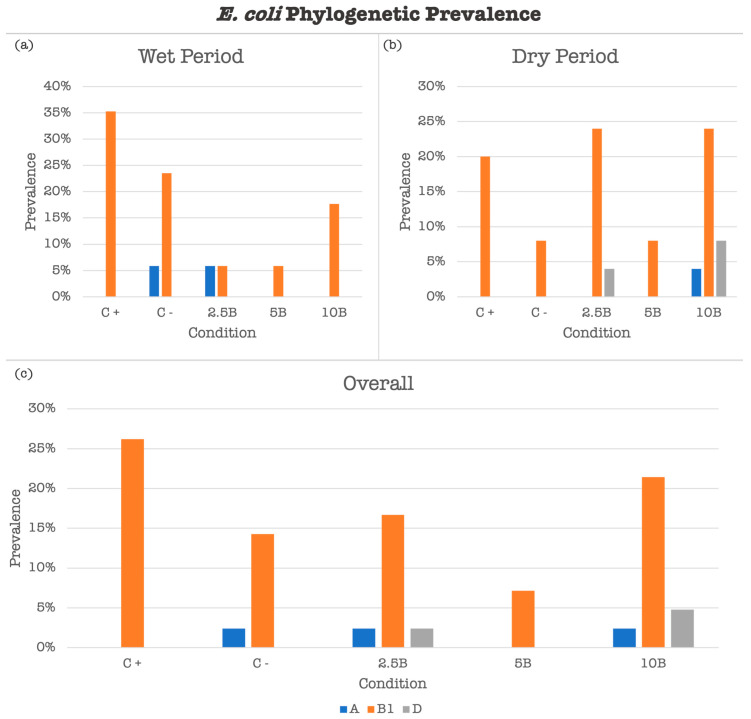
The prevalence of *E. coli* phylogenetic groups. The prevalence of *E. coli* phylogenetic groups (A, B1, D) in RMS samples across different conditions and periods. (**a**) The prevalence of isolates obtained in the wet period (n = 17). (**b**) The prevalence of isolates obtained in the dry period(n = 25). (**c**) The overall resistance rates (n = 42). C+—positive control; C−—negative control; 2.5B—RSM supplemented with 2.5% biochar; 5B—RSM supplemented with 5% biochar; 10B—RSM supplemented with 10% biochar.

The phylogenetic prevalence of *E. coli* isolates in the RMS samples, depicted in Figure 5, shows distinct trends across different conditions and periods.

In the assay performed in the wet period (Figure 5a), isolates from the positive control (C+) condition exhibited the highest prevalence of phylogroup A (40%), followed by phylogroup B1 (5%). In contrast, RMS supplemented with 2.5% biochar (2.5B) had a notable presence of phylogroup B1 (5%), with that of phylogroup A remaining low (5%).

In the assay performed in the dry period (Figure 5b), RMS supplemented with 10% biochar (10B) showed a higher prevalence of phylogroup D (5%) compared to the control conditions. The 2.5% biochar condition also displayed a significant presence of phylogroup B1 (10%).

Overall, the prevalence of phylogroups (Figure 5c) indicates that the positive control (C+) and RMS supplemented with 10% biochar (10B) had the highest occurrences of phylogroup A (25% and 20%, respectively), with phylogroup D being more prevalent in the biochar-treated samples. However, these differences were not statistically significant.

### 3.4. Antimicrobial Susceptibility Testing

Table 4 presents the antimicrobial resistance (AMR) profile of *Enterococcus* isolates from RMS supplemented with varying concentrations of biochar (2.5%, 5%, and 10%), alongside the positive (C+) and negative (C−) controls, across different antibiotic classes.

The key findings indicate that for penicillins, resistance to ampicillin was the highest in the 10% biochar group (55%), followed by the 2.5% biochar group (30%), with no resistance being detected in the positive control. No resistance was observed for amoxicillin–clavulanic acid across all conditions. For glycopeptides, vancomycin resistance was 100% in the positive control, whereas the 10% biochar group showed the highest resistance (9%) among the supplemented groups. In the case of tetracyclines, the highest resistance to oxytetracycline was observed in the 5% biochar group (40%), with no resistance being detected in the negative and positive control groups. Aminoglycosides showed no resistance to high-dose gentamycin across all conditions. For fluoroquinolones, the highest resistance to enrofloxacin was observed in the 10% biochar group (36%), with no resistance being found in the positive control.

The enterococci from the RMS samples supplemented with 5% biochar exhibited the lowest overall resistance rates for this bacterial group, being primarily resistant to oxytetracycline (40%). However, the differences observed in the resistance rates demonstrated by these bacteria to various antibiotics were not statistically significant.

According to Table 5, the AMR profiles of *E. coli* isolates varied across the conditions tested. Regarding penicillins, resistance to ampicillin appeared to be higher in the isolates from the positive (36%) and negative controls (29%) when compared to those from the biochar-supplemented groups. In fact, 11% of the isolates from the samples supplemented with 2.5% biochar showed resistance to this antibiotic, while 8% of those from the samples supplemented with 10% biochar presented a resistance profile. Also, none of the isolates from the samples supplemented with 10% biochar were resistant to ampicillin. Additionally, no resistance to amoxicillin–clavulanic acid was observed in any of the conditions tested. However, the observed differences in resistance rates were not statistically significant.

According to the classification presented by Magiorakos et al. (2012), no isolate was considered multidrug resistant (MDR) [45]

### 3.5. Virulence Assays

Regarding enterococci’s ability to produce virulence factors, biochar supplementation did not promote a reduction in virulence factors’ production by RMS isolates, and the changes that occurred were not statistically significant (Table 6). For *Enterococcus* spp., hemolysin production was the highest in the positive control group (100%), followed by the 5% biochar group (80%). Biofilm production was the most prevalent in the 5% biochar group (60%), followed by the negative control group (45%). Proteinase production peaked in the 10% biochar group (82%). Gelatinase, DNase, and lecithinase were not detected in any of the conditions.

For *E. coli*, hemolysin production was uniformly high at 100% across all conditions. Biofilm production was the highest in the negative control group (57%), followed by the positive control group (36%). Gelatinase, DNase, proteinase, and lecithinase production were not detected in any conditions.

Table 7 presents the distribution of hemolysin production across different *Enterococcus* species isolated from RMS samples. *E. gallinarum* and *Enterococcus* sp. isolates exhibited the highest percentages of hemolysin production (33% and 30%, respectively), while lower percentages were observed for *E. faecalis* (11%), *E. faecium* (7%), and *E. hirae* (4%).

A statistical analysis of antibiotic resistance and virulence factors was conducted to understand the impact of the bacterial group and biochar condition on these parameters. The results are summarized in Table 8.

Regarding antibiotic resistance, the statistical analysis revealed that *E. coli* presented increased odds (3.10) of being resistant to ampicillin in comparison to enterococci. However, no significant differences were found regarding antimicrobial resistance profiles and different biochar supplementations (*p* = 0.43). No significant differences were found across any of the tested variables regarding resistance to other antibiotics.

Also, regarding virulence factors, no significant differences were found regarding any of the variables tested.

### 3.6. Isolates’ Pathogenicity Potential—MAR and Virulence Indexes

The MAR and VIR indexes were calculated for each *Enterococcus* spp. and *E. coli* isolates across different biochar supplementation conditions. The average (AVG) and standard deviation (STD) values for each condition are summarized in Table 9.

The MAR results were not statistically significant, while the VIR index showed a statistical trend for the relation between condition and bacteria (*p* = 0.10), since the isolates from the positive control and from the RMS supplemented with 2.5% biochar had a significantly lower VIR index than those from the RMS supplemented with 5% and 10% biochar. The MAR values indicate that *Enterococcus* isolates from the positive control exhibited the highest resistance, whereas isolates from the RMS supplemented with 5% biochar showed the lowest resistance. The VIR values for *Enterococcus* spp. were the highest amongst the isolates from the RMS supplemented with 5% biochar.

For *E. coli*, the highest MAR value was observed in the negative control, while the VIR values were relatively similar across different conditions, with the highest values being observed in the isolates from the negative control. The 5% biochar supplementation showed the lowest virulence index.

Moreover, only isolates from the positive control group met the criteria for being classified as a low threat, indicating a lower pathogenicity potential. In contrast, isolates from all other conditions were classified as posing no threat based on the averages of their MAR and virulence indexes.

## 4. Discussion

Understanding the dynamics of bacterial populations in recycled manure solids (RMS) is crucial for improving animal and environmental health. Previous findings by Hutchison et al. (2005), Gurtler et al. (2018), and Rapp et al., (2023) demonstrate significant pathogen reductions in manure when using aerobic digestion processes [46,47,48]. The significant reduction in bacterial counts over time is likely due to aerobic digestion, which promotes the breakdown of organic matter, creating less favorable conditions for the survival of certain bacterial genera. Studies indicate that aerobic digestion, especially during the thermophilic phase, plays a crucial role in reducing organic matter and pathogen load [46,47,48]. High temperatures during this phase favor the proliferation of thermophilic bacteria, such as those from the genera *Bacillus* and *Thermus*. These bacteria are essential in breaking down complex organic compounds, thereby reducing nutrient availability for other bacteria and creating an environment less conducive to their survival [49,50].

The initial degradation processes are carried out by mesophilic organisms. As the temperature rises due to the intense digestive activity of microorganisms, thermophilic populations take over and continue the conversion of organic compounds into carbon dioxide. This active stage of composting is characterized by rapid decomposition, persisting until the organic substrates are depleted. Subsequently, microbial activity declines, leading to a drop in temperature. During the curing phase, mesophilic organisms repopulate the compost, and humid substances accumulate, resulting in mature compost [50].

In our study, not all conditions tested promoted a reduction in the RMS’ bacterial counts, and the decreases observed were not statistically significant. In comparison, Ref. [51] reported a 30% reduction in *E. coli* numbers, equivalent to a 1.79 Log10(CFU/g) reduction from a baseline of 5.98 Log10(CFU/g), when applying pine biochar to poultry manure.

The reductions obtained in the *Enterococcus* spp. counts are comparable to those obtained by Perez-Mercado et al. (2019), who reported a decrease of 4.4 Log10(CFU/g) in *Enterococcus* sp. when using biochar as a filter for farm wastewater [29]. This similarity highlights biochar’s potential in reducing bacterial populations, although achieving statistical significance remains challenging.

A species diversity analysis provided insights into the microbial communities’ composition and richness. The most predominant *Enterococcus* species detected were *E. hirae* and *E. gallinarum*. *E. hirae*, commonly found in plants and cattle, is an indicator of healthy animals because its presence is associated with normal, commensal gut microbiota in cattle, often isolated from bovine feces and manure [52]. *E. hirae* pathogenicity is not as well characterized as for other enterococcal species; however, isolates from this species have been associated with infections mainly in humans, including pyelonephritis, endocarditis, and biliary tract infections [20]. *E. gallinarum* is generally not associated with infections, but there are reports of this species presenting low-level resistance to vancomycin, which is concerning as vancomycin is often used as a last-resort antibiotic for treating serious infections caused by Gram-positive bacteria [53,54]. The emergence of vancomycin-resistant enterococci (VRE) can lead to limited treatment options and has significant public health implications. *Enterococcus* species with higher health risks, such as *E. faecium* and *E. faecalis*, were identified in the negative control and RMS supplemented with 5% and 10% biochar. It is important to note that only four isolates from each condition and time point were selected for further characterization. This selection was carried out randomly, which may have influenced the variation in species identified in the control and treatment groups.

For *E. coli*, phylogenetic group D was observed exclusively in isolates from 2.5% and 10% biochar-supplemented RMS. Phylogenetic group D *E. coli* strains are particularly concerning because they are often associated with extraintestinal infections in humans, such as urinary tract infections, sepsis, and neonatal meningitis [35,55]. Additionally, these strains can harbor multiple virulence factors and antibiotic resistance genes, making infections difficult to treat. In animals, phylogroup D *E. coli* can cause various diseases, including colibacillosis in poultry and mastitis in dairy cattle [17,56,57]. The presence of these potentially pathogenic strains in biochar-supplemented RMS highlights the need for a further evaluation of biochar’s impact on microbial communities and its potential to influence pathogen prevalence.

The clonal analysis of both *Enterococcus* and *E. coli* isolates from RMS supplemented with biochar revealed some insights into the microbial community dynamics in dairy farming environments. Biochar supplementation does not appear to be crucial for the establishment of specific strains. Common to both bacterial groups is the presence of clones across different treatments and conditions, possibly representing the most persistent strains.

Overall, although no statistically significant differences were found between treatments, the variations between microbial populations suggest that biochar supplementation could influence the microbial community.

The structure of biochar provides a surface for microbial attachment, and its chemical properties could affect nutrient availability and microbial competition. In spite of the fact that the same batch of biochar was used in all assays, the different concentrations of biochar used for supplementation may have created micro-environments within the RMS that favored certain microbial species over others.

Analyzing bacterial AMR and virulence profiles offered deeper insights into the pathogenicity potential of these microorganisms. Enterococci from RMS samples supplemented with 5% biochar exhibited the lowest overall resistance rates, though resistance to oxytetracycline was notable at 40%. Similarly, *E. coli* showed intermediate resistance to ampicillin and ceftiofur at 33%. Studies have demonstrated that up to 20% of the administrated dose of oxytetracycline can be excreted by dairy cattle feces, which substantially contributes to its presence in manure [1]. These findings suggest that the high excretion rates of oxytetracycline may contribute to the resistance patterns observed, as the antibiotic’s presence in manure provides selective pressure that promotes the development and persistence of resistant bacterial populations [58]. This high excretion rate results in elevated concentrations of oxytetracycline residues in manure, making it one of the most prevalent resistances detected [59]. However, while high excretion rates of oxytetracycline contribute to its presence in manure and may promote resistant bacteria populations, direct correlations between antibiotic use and resistance are complex and influenced by multiple factors, including environmental conditions, bacterial strain variability, and gene transfer mechanisms [59]. Some resistance to other antibiotics was also observed, but none of the isolates met the criteria for multidrug resistance (MDR).

Other studies have demonstrated biochar’s antimicrobial properties in various contexts [23,24,26,27,60]. For example, Jang and Kan (2022) found that biochar can effectively remove various ARGs, though some, such as *tetO* and *ermB*, may persist. Jauregi et al., (2023) reported that composting manure with 5% biochar increased the removal rate of specific ARGs, such as those encoding resistance to sulphonamides and tetracyclines [60,61]. This aligns with our findings, particularly the absence of resistance to trimethoprim–sulfamethoxazole in any condition except an intermediate resistance found in the negative control.

While biochar’s effects on soil microbial diversity and antibiotic resistance have been studied [23,24,60,61], its influence on bacterial virulence potential remains less understood. Our findings show that biochar supplementation did not significantly reduce the production of virulence factors by bacterial isolates. Yan et al. (2023) previously suggested that biochar might enhance microbial quorum sensing and biofilm formation, potentially improving cell viability and communication [62]. For *Enterococcus*, only isolates from RMS samples supplemented with 2.5% biochar exhibited a lower virulence factor prevalence than those from the negative control, specifically regarding the production of biofilm (40%) and proteinase (30%). *E. coli* isolates from RMS supplemented with 5% biochar showed a lower virulence, with hemolysin being the only virulence factor detected. Regarding *Enterococcus* species, *E. gallinarum* and *Enterococcus* sp. exhibited the highest proportions of hemolysin-positive isolates, which might suggest a higher potential for pathogenicity. In contrast, *E. faecalis*, *E. faecium*, and *E. hirae*, although traditionally associated with high pathogenicity in clinical contexts, demonstrated significantly lower rates of hemolysin production. This finding might reflect species-specific variations in virulence factor expression or differences in environmental adaptation.

The pathogenic potential of bacterial isolates, assessed using MAR and virulence indexes, indicated that none of the isolates were classified as a high or moderate threat.

One important limitation of the present study is that it focused on two bacterial groups used as indicators for antimicrobial resistance dissemination, lacking a metagenomic approach. As such, in the future, such an approach should be implemented on a larger number of samples collected at different seasons, aiming to better understand the effect of biochar supplementation on the resistome and virulome of RMS.

## 5. Conclusions

Considering animal and environmental health, this study aimed to evaluate the potential promising role of biochar in tackling AMR and virulent strains present in RMS. However, supplementation with different concentrations of biochar did not result in significant differences regarding bacterial loads or the presence of resistant and virulent bacteria in RMS.

The limitations of this study include its sampling design, with manure collected only twice—once in a wet period and once in a dry period—making it impossible to assess temporal or seasonal effects robustly. Additionally, the focus on *Enterococcus* spp. and *E. coli* limits the conclusions that can be drawn, and in the future, a metagenomic approach should be applied to provide a broader understanding of the influence of RMS supplementation with biochar, including in its resistome and virulome.

In conclusion, while the concentrations of biochar tested did not result in significant changes in bacterial loads nor the presence of resistant and virulent bacteria in RMS, this study provides an initial exploration of the potential of RMS supplementation with pine biochar. Further studies with larger sample sizes, expanded sampling points, and broader microbiological analyses are required to better evaluate biochar’s role in improving RMS microbial safety, aiming at its application in agricultural systems.

## Figures and Tables

**Table 1:** Primers used for *Enterococcus* identification at genus and species levels and in fingerprinting and those used for *E. coli* fingerprinting and phylogroup identification.

Identification	Primer		Product Length	Reference
*Enterococcus*				
*Enterococcus* spp.	Ent1	5′ TACTGACAAACCATTCATGATG 3′	112 bp	[32]
	Ent 2	5′ AACTTCGTCACCAACGCGAAC 3′		
*E. faecium*	FM1	5′ GAAAAAACAATAGAAGAATTAT 3′	215 bp	[33]
	FM2	5′ TGCTTTTTTGAATTCTTCTTTA 3’		
*E. faecalis*	FL1	5’ ACTTATGTGACTAACTTAACC 3’	360 bp	
	FL2	5’ TAATGGTGAATCTTGGTTTGG 3’		
*E. hirae*	HI1	5’ CTTTCTGATATGGATGCTGTC 3’	187 bp	
	HI2	5’ TAAATTCTTCCTTAAATGTTG 3’		
*E. durans*	DU1	5’ CCTACTGATATTAAGACAGCG 3’	295 bp	
	DU2	5’ TAATCCTAAGATAGGTGTTTG 3’		
*E. casseliflavus*	CA1	5’ TCCTGAATTAGGTGAAAAAAC 3’	288 bp	
	CA2	5′ GCTAGTTTACCGTCTTTAACG 3′		
*E. cecorum*	CE1	5′ AAACATCATAAAACCTATTTA 3′	371 bp	
	CE2	5′ AATGGTGAATCTTGGTTCGCA 3′		
Fingerprinting	(GTG)_5_	5′ GTGGTGGTGGTGGTG 3′	200–3000 bp	[34]
*E. coli*				
Fingerprinting	ERIC2	5′ AAGTAAGTGACTGGGGTGAGCG 3′	380–3280 bp	[35]
*gadA*	Forward	5′ GATGAAATGGCGTTGGCGCAAG 3′	373 bp	[36]
	Reverse	5′ GGCGGAAGTCCCAGACGATATCC 3′		
*chuA*	Forward	5′ ATGATCATCGCGGCGTGCTG 3′	281 bp	
	Reverse	5′ AAACGCGCTCGCGCCTAAT 3′		
*yjaA*	Forward	5′ TGTTCGCGATCTTGAAAGCAAACGT 3′	216 bp	
	Reverse	5′ ACCTGTGACAAACCGCCCTCA 3′		
TSPE4.*C2*	Forward	5′ GCGGGTGAGACAGAAACGCG 3′	152 bp	
	Reverse	5′ TTGTCGTGAGTTGCGAACCCG 3′		

**Table 2:** A summary of comparisons for *Enterococcus* spp. and *Enterobacteriaceae* counts. This table presents post hoc comparisons of the log-transformed bacterial counts for both *Enterococcus* spp. and *Enterobacteriaceae* across different treatments and time points. Significant comparisons are highlighted in green, demonstrating the effects of time on bacterial reduction.

Comparisons	*Enterococcus* spp. *p*-Value	*Enterobacteriaceae* *p*-Value
Day 0 vs. Day 5	<0.0001	0.9715
Day 0 vs. Day 15	<0.0001	0.9436
Day 0 vs. Day 30	<0.0001	0.4090
Negative Control vs. 2.5% Biochar	0.7346	0.8808
Negative Control vs. 5% Biochar	0.1156	0.9951
Negative Control vs. 10% Biochar	0.3881	0.9967

**Table 3:** The species distribution (%) of *Enterococcus* in RMS for each condition. The overall percentage represents the sum of isolates for both periods divided by the total number of isolates for both periods. C+—positive control; C−—negative control; 2.5B—RSM supplemented with 2.5% biochar; 5B—RSM supplemented with 5% biochar; 10B—RSM supplemented with 10% biochar.

	Period	C+	C−	2.5B	5B	10B
*E. faecium*	Wet	0	16	0	3	6
	Dry	0	0	0	0	0
	Overall	0	10	0	2	4
*E. faecalis*	Wet	0	0	0	6	0
	Dry	0	0	0	0	0
	Overall	0	0	0	4	0
*E. durans*	Wet	0	0	0	0	0
	Dry	0	0	0	0	0
	Overall	0	0	0	0	0
*E. hirae*	Wet	0	10	0	0	3
	Dry	0	5	32	11	5
	Overall	0	6	12	4	4
*E. gallinarum*	Wet	0	0	13	0	16
	Dry	16	5	5	0	0
	Overall	6	0	10	0	10
*Enterococcus* sp.	Wet	0	10	6	6	3
	Dry	0	11	0	0	11
	Overall	0	8	6	4	6

**Table 4:** Results from antimicrobial susceptibility tests for *Enterococcus* isolates (n_total_ = 40). Isolates across all time points for each condition with corresponding susceptibility and resistance rates for each antibiotic tested. R (%): percentage of resistant isolates; I (%): percentage of intermediate isolates; S (%): percentage of susceptible isolates. N/A—no breakpoints available.

		Penicillin’s		Glycopeptides	Tetracyclines	Aminoglycosides	Fluoroquinolones
	Condition	Ampicillin	Amoxicillin–Clavulanic Acid	Vancomycin	Oxytetracycline	High-Dose Gentamycin	Enrofloxacin
%R	C+ (n = 3)	0	0	66.7	0	0	0
	C− (n = 11)	27.3	0	0	0	0	18.2
	2.5B (n = 10)	30	0	0	0	0	10
	5B (n = 5)	0	0	0	40	0	0
	10B (n = 11)	54.5	0	9.1	18.2	0	36.4
%I	C+ (n = 3)	N/A	0	33.3	0	0	100
	C− (n = 11)	N/A	0	9.1	18.2	0	36.4
	2.5B (n = 10)	N/A	0	40	10	0	50
	5B (n = 5)	N/A	0	20	0	0	0
	10B (n = 11)	N/A	0	9.1	18.2	0	9.1
%S	C+ (n = 3)	100	100	0	100	100	0
	C− (n = 11)	72.7	100	90.9	81.8	100	45.5
	2.5B (n = 10)	100	100	60	90	100	40
	5B (n = 5)	100	100	80	60	100	100
	10B (n = 11)	45.5	100	81.8	63.6	100	54.5

**Table 5:** Results from antimicrobial susceptibility tests for *E. coli* (n_total_ = 42). Isolates across all time points for each condition with corresponding susceptibility and resistance rates for each antibiotic tested. R (%): percentage of resistant isolates; I (%): percentage of intermediate isolates; S (%): percentage of susceptible isolates. Results in bold represent highest %R for each antibiotic.

		Penicillin’s		Tetracyclines	Sulphonamides	Fluoroquinolones	Cephalosporins
	Condition	Ampicillin	Amoxicillin–Clavulanic Acid	Oxytetracycline	Trimethoprim– Sulfamethoxazole	Enrofloxacin	Ceftiofur
%R	C+ (n = 11)	36.4	0.0	0.0	0.0	0.0	0.0
	C− (n = 7)	28.6	0.0	14.3	0.0	0.0	0.0
	2.5B (n = 9)	11.1	0.0	11.1	0.0	0.0	0.0
	5B (n = 3)	0.0	0.0	0.0	0.0	0.0	0.0
	10B (n = 12)	8.3	0.0	16.7	0.0	0.0	0.0
%I	C+ (n = 11)	36.4	0.0	0.0	0.0	0.0	0.0
	C− (n = 7)	28.6	14.3	14.3	14.3	0.0	0.0
	2.5B (n = 9)	33.3	0.0	0.0	0.0	0.0	11.1
	5B (n = 3)	33.3	0.0	0.0	0.0	0.0	33.3
	10B (n = 12)	41.7	0.0	0.0	0.0	0.0	0.0
%S	C+ (n = 11)	27.3	100.0	100.0	100.0	100.0	100.0
	C− (n = 7)	42.9	85.7	71.4	85.7	100.0	100.0
	2.5B (n = 9)	55.6	100.0	88.9	100.0	100.0	88.9
	5B (n = 3)	66.7	100.0	100.0	100.0	100.0	66.7
	10B (n = 12)	50.0	100.0	83.3	100.0	100.0	100.0

**Table 6:** Virulence profiles of *Enterococcus* (n_total_ = 40) and *E. coli* (n_total_ = 42). Isolates across all time points for each condition. *p* (%): percentage of positive isolates.

		Condition	Hemolysin	Gelatinase DNase Lecithinase	Biofilm	Proteinase
*Enterococcus*	%P	C+ (n = 3)	100	0	0	0
		C− (n = 11)	55	0	45	55
		2.5B (n = 10)	60	0	40	30
		5B (n = 5)	80	0	60	80
		10B (n = 11)	73	0	36	82
*E. coli*	%P	C+ (n = 11)	100	0	36	0
		C− (n = 7)	100	0	57	0
		2.5B (n = 9)	100	0	11	0
		5B (n = 3)	100	0	0	0
		10B (n = 12)	100	0	33	0

**Table 7:** Hemolysin production (%) across *Enterococcus* species isolated from RMS samples.

	Hemolysin
*E. faecalis*	11%
*E. faecium*	7%
*E. hirae*	4%
*E. gallinarum*	33%
*Enterococcus* sp.	30%

**Table 8:** A statistical analysis of antibiotic resistance and virulence factors. This table presents the *p*-values (significant *p*-value < 0.05) from the statistical analysis of the effects of the bacterial group and biochar condition on antibiotic resistance and virulence factors. Highlighted in gray are the results of the analysis performed for only one bacterial group. Highlighted in yellow are all isolates that presented the same phenotype. The dash (-) represents variables not included in the final models.

Antibiotic/Virulence Factor	Bacteria (*p*-Value)	Condition (*p*-Value)
Ampicillin	0.03	0.43
Oxytetracycline	-	0.80
Trimethoprim-Sulfamethoxazole		1.00
Enrofloxacin	-	0.92
Amoxicillin-clavulanic acid	-	1.00
Ceftiofur		0.95
Vancomycin		0.60
Gentamicin		-
Hemolysin	-	0.51
Gelatinase	-	-
Biofilm	-	0.63
DNase	-	-
Lecithinase	-	-
Proteinase	-	0.45

**Table 9:** MAR and VIR averages for each treatment condition. AVG—average; STD—standard deviation. Results in bold show values above cut-off. For each parameter, means with different superscript letters indicate significant differences between groups.

		C+		C−		2.5B		5B		10B	
		AVG	STD	AVG	STD	AVG	STD	AVG	STD	AVG	STD
*Enterococcus*	MAR	**0.33**	0.00	0.18	0.12	0.23	0.25	0.10	0.15	0.26	0.11
	VIR	0.17 ^a^	0.00	0.26 ^ab^	0.16	0.22	0.11	0.37 ^b^	0.14	0.32 ^b^	0.12
*E. coli*	MAR	0.12	0.08	0.19	0.24	0.11	0.08	0.11	0.10	0.11	0.08
	VIR	0.23	0.08	0.26	0.09	0.19	0.06	0.17	0.00	0.22	0.08

## Data Availability

Data is contained within the article and Supplementary Materials.

## Supplementary Materials

The following supporting information can be downloaded at https://www.mdpi.com/article/10.3390/vetsci12010043/s1, Table S1: A BLAST comparison analysis of PCR product sequences obtained from *Enterococcus* isolates. The table lists the primers used, sequences obtained, product lengths, and the percentage identification for each sequence, confirming the species identification as *E. gallinarium*, Table S2: *Enterobacteriaceae* quantification in CFU/g and [Log10(CFU/g)] in MAC inoculated with samples collected during the assays performed in the dry and wet periods, Table S3: *Enterococcaceae* quantification in CFU/g and [Log10(CFU/g)] in SB inoculated with samples collected during the assays performed in the dry and wet periods.
