# Supplementary material for: Potential of Pine Biochar to Mitigate Bacterial Hazards Present in Recycled Manure Solids from Dairy Cows
Source: Vet Sci. 2025 Jan 10;12(1):43. doi: 10.3390/vetsci12010043 (PMC11769459; doi:10.3390/vetsci12010043)
Supplement: Supplementary file 1 [file vetsci-12-00043-s001.zip › vetsci-3237066-supplementary.pdf]

Supplementary Table

Table S1 - BLAST comparison analysis of PCR product sequences obtained from *Enterococcus* isolates. The table lists the primers used, sequences obtained, product lengths, and the percentage identification for each sequence, confirming the species identification as *E. gallinarum*.

Primer used	Sequence Obtained	Product Length	% Identification
CA1	TGCTGATTTTGATTCGGTGCCTGAAGA<br>CATCAAAACAGCTGTCCGTAATAACG<br>GTGGTGGTCATGCAAATCACAGCTTTT<br>TCTGGGAAATCTTGGCACCAAATGCTG<br>GTGGTGAACCAACAGGAGCCATCAAA<br>GAAGCCATCGAAGAAACATTTGGCAG<br>CTTTGCTGATTTCAAAGAAGAATTCAA<br>AACAGCAGCAACTGGCCGCTTTGGTT<br>CTGGCTGGGCTTGGTTAGTCATCAAAG<br>ATGGTAAATTAGCGATCACTTCAACTG<br>CGAACCAAGAATTACACCATTAAA	290 bp	99.31%
CE2	GCATGACTAACCAAGCCCAGCCAGAA<br>CCAAAGCGGCCAGTTGCTGCTGTTTTG<br>AATTCTTCTTTGAAATCAGCAAAGCTG<br>CCAAATGTTTCTTCGATGGCTTCTTTGA<br>TGGCTCCTGTTGGTTCACCACCAGCAT<br>TTGGTGCCAAGATTTCCCAGAAAAAG<br>CTGTGATTTGCATGACCACCACCGTTA<br>TTACGGACAGCTGTTTTGATGTCTTCA<br>GGCACCGAATCAAAATCAGCAAGTAA<br>TTCTTCAACTGTTTTTTCACCTAATTCA<br>GGAAAT	275 bp	99.63%

Table S2 - *Enterobacteriaceae* quantification in CFU/g and [Log10(CFU/g)] in MAC inoculated with samples collected during the assays performed in the dry and wet seasons.

Enterobacteriaceae

Season	Time Point	Replicate	Negative Control		RMS + 2.5% Biochar		RMS + 5% Biochar		RMS + 10% Biochar	
			CFU/g	Log10(CFU/g)	CFU/g	Log10(CFU/g)	CFU/g	Log10(CFU/g)	CFU/g	Log10(CFU/g)
Dry	D0	R1	1.00E+07	7.00	1.28E+07	7.11	1.30E+07	7.11	1.80E+07	7.26
		R2	1.90E+07	7.28	6.10E+06	6.79	5.00E+06	6.70	5.90E+06	6.77
		R3	1.30E+07	7.11	1.64E+07	7.21	6.00E+06	6.78	2.90E+06	6.46
	D5	R1	2.00E+04	4.30	1.60E+06	6.20	1.00E+06	6.00	1.60E+06	6.20
		R2	1.70E+06	6.23	2.40E+06	6.38	5.60E+06	6.75	1.60E+06	6.20
		R3	8.60E+05	5.93	3.00E+05	5.48	1.30E+06	6.11	1.50E+06	6.18
	D15	R1	1.56E+06	6.19	7.30E+05	5.86	4.60E+06	6.66	7.40E+05	5.87
		R2	7.30E+05	5.86	5.70E+05	5.76	2.00E+05	5.30	7.40E+05	5.87
		R3	1.15E+06	6.06	6.50E+05	5.81	4.00E+05	5.60	7.40E+05	5.87
	D30	R1	8.00E+06	6.90	5.20E+07	7.72	1.05E+07	7.02	4.30E+06	6.63
		R2	1.00E+07	7.00	1.90E+07	7.28	7.00E+05	5.85	5.00E+05	5.70
		R3	4.00E+06	6.60	6.40E+07	7.81	6.50E+06	6.81	9.70E+06	6.99
Wet	D0	R1	1.41E+06	6.15	4.10E+05	5.61	6.00E+05	5.78	5.00E+05	5.70
		R2	1.14E+06	6.06	4.10E+05	5.61	3.00E+05	5.48	5.30E+06	6.72
		R3	1.28E+07	7.11	4.10E+05	5.61	5.00E+05	5.70	9.00E+05	5.95
	D5	R1	1.00E+07	7.00	2.00E+07	7.30	2.50E+07	7.40	1.20E+08	8.08
		R2	5.00E+06	6.70	3.90E+07	7.59	2.10E+07	7.32	3.00E+06	6.48
		R3	8.00E+06	6.90	0.00E+00	0.00	1.70E+07	7.23	2.37E+08	8.37
	D15	R1	4.00E+06	6.60	3.00E+06	6.48	1.50E+07	7.18	2.50E+06	6.40
		R2	7.80E+06	6.89	2.30E+07	7.36	1.11E+07	7.05	5.70E+06	6.76
		R3	1.06E+07	7.03	1.10E+07	7.04	2.00E+06	6.30	5.20E+06	6.72
	D30	R1	2.20E+07	7.34	6.30E+07	7.80	1.30E+07	7.11	7.80E+06	6.89
		R2	2.20E+07	7.34	1.10E+07	7.04	4.00E+06	6.60	8.30E+06	6.92
		R3	2.20E+07	7.34	2.50E+07	7.40	2.20E+07	7.34	1.80E+06	6.26

Table S3 – *Enterococcaceae* quantification in CFU/g and [Log10(CFU/g)] in MAC inoculated with samples collected during the assays performed in the dry and wet seasons.

Enterococcaceae										
**Season**	**Time Point**	**Replicate**	**Negative Control**		**RMS + 2.5% Biochar**		**RMS + 5% Biochar**		**RMS + 10% Biochar**	
			CFU/g	Log10(CFU/g)	CFU/g	Log10(CFU/g)	CFU/g	Log10(CFU/g)	CFU/g	Log10(CFU/g)
Dry	D0	R1	3.90E+06	6.59	1.70E+06	6.23	1.39E+06	6.14	1.72E+06	6.24
		R2	3.40E+06	6.53	1.75E+06	6.24	1.30E+06	6.11	1.63E+06	6.21
		R3	1.30E+06	6.11	1.32E+06	6.12	1.10E+06	6.04	5.40E+05	5.73
	D5	R1	1.10E+04	4.04	6.00E+03	3.78	2.00E+03	3.30	7.10E+04	4.85
		R2	9.00E+03	3.95	1.80E+04	4.26	5.10E+04	4.71	1.30E+04	4.11
		R3	6.00E+03	3.78	1.60E+04	4.20	1.62E+05	5.21	2.40E+04	4.38
	D15	R1	0.00E+00	0.00	4.00E+03	3.60	0.00E+00	0.00	0.00E+00	0.00
		R2	7.00E+03	3.85	0.00E+00	0.00	0.00E+00	0.00	0.00E+00	0.00
		R3	0.00E+00	0.00	0.00E+00	0.00	0.00E+00	0.00	0.00E+00	0.00
	D30	R1	8.00E+03	3.90	0.00E+00	0.00	0.00E+00	0.00	0.00E+00	0.00
		R2	0.00E+00	0.00	0.00E+00	0.00	0.00E+00	0.00	0.00E+00	0.00
		R3	0.00E+00	0.00	0.00E+00	0.00	0.00E+00	0.00	0.00E+00	0.00
Wet	D0	R1	5.10E+05	5.71	3.70E+05	5.57	1.50E+05	5.18	5.60E+05	5.75
		R2	3.60E+05	5.56	2.50E+05	5.40	4.60E+05	5.66	1.50E+05	5.18
		R3	1.00E+06	6.00	2.20E+05	5.34	8.00E+04	4.90	2.70E+05	5.43
	D5	R1	3.00E+03	3.48	9.00E+03	3.95	1.00E+04	4.00	3.00E+04	4.48
		R2	1.30E+04	4.11	3.70E+04	4.57	3.60E+04	4.56	1.80E+04	4.26
		R3	4.10E+04	4.61	4.00E+03	3.60	6.00E+03	3.78	2.00E+04	4.30
	D15	R1	1.00E+03	3.00	5.20E+04	4.72	0.00E+00	0.00	1.00E+03	3.00
		R2	0.00E+00	0.00	0.00E+00	0.00	0.00E+00	0.00	0.00E+00	0.00
		R3	0.00E+00	0.00	0.00E+00	0.00	0.00E+00	0.00	0.00E+00	0.00
	D30	R1	0.00E+00	0.00	0.00E+00	0.00	0.00E+00	0.00	0.00E+00	0.00
		R2	1.00E+03	3.00	0.00E+00	0.00	0.00E+00	0.00	0.00E+00	0.00
		R3	0.00E+00	0.00	0.00E+00	0.00	0.00E+00	0.00	0.00E+00	0.00
